# Understanding Intimate Partner Violence Among Ethnic and Sexual Minorities: Lived Experiences of Queer Women in Norway

**DOI:** 10.1177/10778012221147912

**Published:** 2022-12-29

**Authors:** Esra Ummak, Salman Turken, Deniz Akin

**Affiliations:** 1 Department of Social Work, Faculty of Social Sciences, 87368 VID Specialized University, Oslo, Norway; 2 Early Childhood Education, 60499 Oslo Metropolitan University, Oslo, Norway; 3 Department of Interdisciplinary Studies of Culture, Eastern Norway Research Institute, Inland Norway University of Applied Sciences & NTNU, Trondheim, Norway

**Keywords:** intimate partner violence, queer women, intersectionality, minority stress, ethnic minorities

## Abstract

Drawing upon in-depth, semi-structured interviews, this study explored how queer women from ethnic minority backgrounds in Norway understand and experience intimate partner violence (IPV). Based on an intersectional approach, the study highlights and discusses how having multiple minority positions may inform and affect the way participants experience IPV. The analysis shows that participants’ experiences of IPV are shaped by their multiple minority statuses in Norwegian society. A discussion is provided that revolves around how being a sexual as well as an ethnic minority generates a significant power imbalance for the participants in their relationships.

The World Health Organization (WHO) categorizes intimate partner violence (IPV) as a global health concern that causes severe and negative mental and physical health outcomes among both heterosexual and queer individuals (WHO, 2010). IPV has been proposed to include “behaviour within an intimate relationship that causes physical, sexual or psychological harm, including acts of physical aggression, sexual coercion, psychological abuse and controlling behaviours” (Heise & Garcia-Moreno, 2002, p. 89). It may also take the form of material and economic violence, a type of control over a partner's “ability to acquire, use and maintain economic resources, thus threatening her economic security and potential for self-sufficiency” (Adams et al., 2008, p. 564; see also Norwegian Directorate for Children, Youth and Family Affairs [Bufdir], 2021). IPV may further be understood as behavior that “instils fear or offense, and in this way makes the person to do something against her will, or to discontinue doing something that is in accordance with her will” (Isdal, 2000, p. 36), thus reducing the autonomy of the person. While research on IPV in heterosexual couples reveals a large variance in prevalence across cultures (WHO, 2010), the increasing, albeit still few, number of studies on IPV in queer relationships indicate a higher prevalence of IPV among queer women, especially compared to their heterosexual counterparts (e.g., Blosnich & Bossarte, 2009; Messinger, 2011).

Social policies regarding IPV influenced by the still-prominent second-wave Western feminist approach starting in 1970s have attempted to understand violence in general, reflecting and reproducing the heteronormative understandings of society. In this direction, researchers have positioned “men” as the active agents of violence and “women” as passive receivers or sufferers (Millett, 1971). Consequently, the commonly held assumption that IPV stems from the duality of women and men, and that the passive recipients of male violence are the ones who are married women/spouses within the family system, maintains the invisibility of the IPV between queer individuals. Research on IPV thus has been increasingly criticized for its dependence on conceptual binaries such as *man/woman* and *agency/victimhood* (see also Haaken, 2010). Eschewing such duality, we acknowledge and argue that there can be a power imbalance or struggle, constitutive of violence, in queer relationships. Given the increasing visibility of queer forms of relationships, there is a need to increase our knowledge and awareness of the different forms of violence occurring in such relationships.

Although IPV has been an important field of study across disciplines, there is indeed a considerable absence of research that focuses on IPV among queer people (Calton et al., 2016; Stubberud & Eggebø, 2020; West, 2002). As available studies indicate, various types of IPV do occur among partners across gender and sexual identities, and there is an urge for further research to highlight and analyze how sexual minority status shapes violence experiences. Failing to widen the scope when it comes to how we perceive and study IPV puts queer individuals at risk, especially queer individuals with multiple forms of societal marginalization (e.g., queer women from ethnic minority backgrounds) (Fountain & Skolnik, 2007). Therefore, we suggest that IPV among queer individuals should be understood through intersectional lenses to move beyond single-axis explanations of violence and focus on how, for instance, “race,” sexual identity, sexual orientation, immigrant status, and economic status may intersect and inform experiences of IPV.

Accordingly, in this study, we focused specifically on how queer women from minority backgrounds in Norway understand and experience IPV. Queer women in this study included individuals with the gender identity of cis-woman and sexual orientation of lesbian, bisexual, pansexual, demisexual, etc. We also discuss how the cultural politics of immigration in Norway can be important in these personal experiences. Understanding the unique characteristics of IPV among queer women from ethnic minority backgrounds may help identify alternate pathways for research and intervention for members of such marginalized groups. The results can also be beneficial for policy makers in adopting broader and more inclusive measures for protection against IPV.

## IPV in Norway

Experiences of IPV may exacerbate mental health problems in the lesbian, gay, bisexual, transgender, and queer (LGBTQ) population, who have been shown in several studies to suffer from disproportionate rates of mental health problems compared to the general population in multiple countries (Frisch et al., 2019; Mink et al., 2014). In Norway, several studies have documented the negative experiences LGBTQ persons encounter compared to the larger heterosexual population. Roland and Auestad (2009) reported for instance that 36% gay youth had been exposed to mobbing compared to 6% of heterosexual youth. According to Fladmoe et al. (2019), LGBTQ persons reported to a larger degree having experienced hateful comments (23% compared to 10% in the larger population) and concrete threats (15% compared to 4% in the larger population). In addition, Eggebø et al. (2018) reported that LGTBQ persons with immigrant background may be vulnerable to discrimination and stereotyping in complex ways due to their multiple minority identities. Overall, the share of LGBTQ individuals reporting mental health challenges, such as depression and anxiety, substance abuse, and suicidal thoughts or attempts is considerably larger than among heterosexuals. Thirty-six percentage of LGBTQ individuals report symptoms of mental health problems, compared to 18% of the general public (Bufdir, 2021). Furthermore, LGBTQ individuals can be seen as an at-risk group in connection to IPV, in terms of both potential exposure and mental health outcomes. However, in Norway, there have been only a few studies focusing on IPV in the LGBTQ population (Fjær et al., 2013; Hesselberg, 2011). Although these studies document the fact that IPV in LGBTQ population occurs, they provide very little or no information about the prevalence of IPV and salience within LGBTQ population in general and among those with immigrant background in particular. While Stubberud and Eggebø (2020) report various forms of violence queer immigrants in Norway experience, including hate crime, racism, and honor-related violence by family, they acknowledge the lack of research on IPV especially. This lack of knowledge is also pointed out by the Norwegian shadow report to the Istanbul Convention (2020), which states that diversity is poorly addressed in existing violence research in Norway, and additional research on violence against and among LGBTQ individuals has not been completed as planned in the most recent national action plan against violence (Norsk Skyggerapport – Istanbulkonvensjonen, 2020). According to the report, there is a particular lack of data when it comes to mapping queer women's experiences of violence. Thus, the paucity of research on IPV among queer women in Norway is a significant obstacle to creating evidence-based policies, outreach, and programs targeting the issue.

## Minority Stress

The theory of minority stress is a conceptual framework developed to understand stressors embedded in the social position of gender and sexual minorities, and their relation to mental health challenges such as depressive symptoms, suicide ideation, substance abuse, etc. (Meyer, 2003).

From a minority stress theory perspective, queers can experience distress due to the stigma and prejudice based on their gender and sexual identities. Furthermore, minority stress might be experienced differently among queers based on membership of other social identity categories (i.e., race, gender, sexual orientation, etc.). In other words, the variation of experiences of stress within the queer population might be understood by differential stress exposures, such as racism and sexism. In addition to mental health problems, the combined stressors of heterosexism and racism could create unique vulnerabilities for psychosocial challenges, such as IPV (Finneran & Stephenson, 2014).

## Intersecting Identities: Being a Queer Woman of an Ethnic Minority in Norway

The framework of *intersectionality* has been utilized as an analytical perspective to make sense of the multidimensional aspects of oppression and individual experiences in which one simultaneously accommodates several socially constructed minority categories such as race, class, gender, and ethnicity. For instance, Crenshaw (1989), who is credited with coining the term, argued that the existing legal system, at that particular time in the United States, failed to capture how racism and sexism simultaneously contribute to the oppression of black women. Cole (2009), arguing for the importance of understanding the relationship of race/ethnicity, gender, social class, and sexuality on outcomes such as mental health and wellbeing, suggests that psychologists conceptualize the influences of multiple social categories in their research. From an intersectionality perspective, race, gender, social class, sexuality, and other social categories are construed to influence groups’ access to social, economic, and political resources and privileges, as they “encapsulate historical and continuing relations of political, material, and social inequality and stigma” (Cole, 2009, p. 173). In addition, Mahalingam (2007) characterizes intersectionality in terms of the “interplay between person and social location, with particular emphasis on power relations among various social locations” (p. 45), drawing our attention to the ways in which multiple category memberships position individuals and groups in asymmetrical relation to one another. This implies an understanding of individuals as embedded in cultural and historical contexts, which not only may affect the way they perceive and experience life but also structure what outcomes are available to them.

While LGBTQ status is socially stigmatized in societies that have predominantly heteronormative cultures, other social statuses (e.g., race/ethnicity, gender) can interact with it to create increasingly unequal mental health outcomes (Mink et al., 2014; Khan et al., 2017). Within the context of IPV in which the partner is a majority member, it may be expected that having multiple minority identities could compound the effects of discrimination, cultural bias, stigma, and intolerance with the effects of abuse in the intimate sphere. Intersectionality demands that we consider how subjective experiences of structural inequalities and stigma can be constitutive of mental health outcomes among multiple marginalized social groups.

When studying the experiences and understandings of IPV for queer women from ethnic minority backgrounds, we seek to capture how these identities (being an ethnic and sexual minority) intersect and shape the IPV experiences of this particular group in the particular context of Norway. In doing so, we also aim to highlight the multifaceted power dynamics operating in IPV instances that are specific to these minorities. For example, legal regulations and practices regarding the rights of LGBTQ people are advanced in Norway compared to many other nations (Bufdir, 2014). Furthermore, using the current Rainbow Index published by the European Region of the International Lesbian, Gay, Bisexual, Trans, and Intersex Association (ILGA-Europe, 2020), Norway ranks 4th (69.89% on a scale of 0–100) in terms of respect for the human rights of LGBTQ individuals. Nonetheless, some LGBTQ people from ethnic minorities in Norway might feel rejected by the mainstream gay community, where they might face racism and a lack of acceptance of their values and attitudes (Bufdir, 2014). Furthermore, having at least two minority statuses—that is, as women in patriarchal cultures and as queer women in heteronormative societies—may result in augmenting minority stress (Szymanski & Kashubeck-West, 2008). At this point, queer women (e.g., women with a non-normative sexual orientation) are more likely than others to become victims of violence, and ethnic minorities are particularly vulnerable in the context of IPV (Bufdir, 2014).

The lack of research on IPV in queer relationships in general in Norway, with limited exception (e.g., Fjær et al., 2013; Hesselberg, 2011), and the lack of research specifically in queer relationships in which one partner is a minority member (Stubberud & Eggebø, 2020) provide justification to do more research to grasp the phenomenon of IPV in queer relations. Here, we are specifically interested in capturing the lived experiences of queer women from ethnic minority backgrounds in Norway through an intersectional approach to IPV. We aim to widen our understanding and contribute to the research field by using a qualitative approach.

## Methods

Our research orientation was qualitative, and we chose semi-structured interview as the main method of data collection. Kvale (1996) suggests that semi-structured interviews may work as a way of performing a planned yet flexible conversation, which provides the possibility of gathering data about the life worlds of participants and interpreting meanings attached to phenomena described by them. The first author conducted all interviews, which lasted approximately 1.5 h each. All interviews were conducted in English.

Throughout the research process, we discussed how, in addition to epistemological reflexivity, our positionality might inform the study and provide insights to analyzing the data, as suggested by Willig (2013). For instance, all three authors are immigrants in Norway, coming from a Middle Eastern country. Two of the authors identify as queer. To illustrate further, the first author who conducted the interviews experienced to have established the necessary rapport, the comfortable safe space in an interview situation where participants can speak freely and openly (Willig, 2013), with the participants.

## Participants

We recruited nine participants by distributing a poster with information about the study and the criteria for participation through email groups of established networks and NGOs working for LGBTQ rights (such as Skeiv Verden*,* FRI*,* and Salaam) in Norway. Thus, our sample is a convenience sample of only those who were interested in the study got in touch with us. Criteria for selection included (a) volitional participation, (b) age of 18 years or older, (c) identifying as a queer woman (i.e., gender identity of cis-woman and sexual orientation of lesbian, bisexual, pansexual, etc.) who has engaged in relationships with women (i.e., cis, non-binary, trans, etc.), (d) having experienced a violent romantic relationship (i.e., dating, cohabiting, engaged, or married), and (e) having migration background in Norway. We will use the following culturally appropriate pseudonyms for our participants: Esin, Eirinn, Elena, Agnes, Nadiya, Dilan, Sinem, Aru, and Yasemen. Six of participants had immigrated to Norway as adults (length of stay ranging from two to 12 years) except Eirinn, Dilan and Aru, who were born in Norway to immigrant parents. Participants or their families had migrated from countries in Africa, Middle East, and Eastern Europe. All identified themselves as queer women, except Yasemen, who identified as non-binary. The mean age of our participants was 33 years (range: 25–42 years). All had been in at least one romantic relationship in which they experienced IPV (Table 1).

**Table 1.** Participant Characteristics.

	Age	Length of stay in Norway	Migration from	Gender identity	Sexual orientation
Esin	42	12	Middle East	Cis woman	Lesbian
Eirinn	26	26^a^	Africa	Cis woman	Bisexual
Elena	25	2	Middle East	Cis woman	Pansexual
Agnes	40	10	Eastern Europe	Cis woman	Lesbian
Nadiya	38	10	Eastern Europe	Cis woman	Bisexual
Dilan	27	27*	Middle East	Cis woman	Lesbian
Sinem	32	10	Middle East	Cis woman	Pansexual
Aru	29	29^a^	Africa	Cis woman	Demisexual
Yasemen	37	2	Middle East	Non-Binary	Lesbian

a Born to immigrant parents in Norway.

## Procedure

Thematic analysis (TA) as outlined by Braun and Clarke (2006, 2019) is utilized to make sense of the collected data. Doing TA implies looking at data at a latent level, allowing researchers to generate themes: “patterns of shared meaning, underpinned by a central meaning-based concept” (Braun & Clarke, 2019, p. 593) in what is reported by the participants. Reflexivity underpins any methodological choice and relates to “ways in which a researcher's involvement with a particular study influence, acts upon and informs such research” (Nightingale & Cromby, 1999, p. 228). We adopt a constructionist position and aim to interpret and relate data to the most salient discourses, social, and structural elements of a society. Within this position lies an implication of an ontological acceptance of the possibility of multiple socially constructed realities and truth claims, coupled with an epistemological dependence relating to ways in which participants and we as researchers mutually construct meanings (Head & Milton, 2014; see also Willig, 2013).

We followed a stepwise process as suggested by Braun and Clarke (2006). First, interviews were transcribed verbatim and anonymized in line with the ethical standards set forth by the Norwegian Center for Research Data, which approved the conduct of the current study. Second, each coauthor coded three separate interviews on their own, creating an initial code list. Then, the codes we each suggested from this initial phase were compared to those coded by the other two coauthors by gathering codes in a common code book. At this stage, each coauthor read all interviews to become more familiar with the data. We held meetings to generate further codes and or refine the codes from the initial phase. After through discussions regarding the data and our positionality (our immigrant background, sexual identity and orientation, and academic and epistemological considerations), we reached a consensus about the common themes, which are reported in the next section.

## Results

In line with our research questions, we will first present how our participants understand and define IPV. We will then turn to ways in which they make sense of their IPV experiences, also detailing from an intersectional perspective how their experiences are necessarily embedded within and permeated and informed by the wider social and societal context (Table 2).

**Table 2.** Defining Intimate Partner Violence.

Type of violence	Example quote
Physical	“Hitting and pushing against the wall.” (Agnes, 40)
Sexual	“Transmitting sexual disease to a partner or having unprotected sex with others while hiding it from one's partner.” (Eirinn, 26)
Economic	“Deny the partner access to their own money.” (Esin, 42)
Psychological	“Degrading, putting down, and making feel worthless.” (Sinem, 32)

### Defining IPV

A broad conceptualization of violence was a common thread in the way our participants understood and talked about IPV. Within this broad conceptualization, they illustrated four forms of abuse that they had experienced related to IPV: physical, sexual, economic, and psychological. Elena, one of the participants, defines IPV as “basically anything that is done by a partner toward the other partner to show dominance in terms of relationship standard.” We should emphasize that although these four forms of abuse are conceptually distinct and thus are categorized in our analysis as different themes, they might, from our participants’ point of view, be *experientially* overlapping. As Eirinn illustrates, the line between different forms of abuse might not always be clear-cut: “during sex or whatever, she would scratch me so hard, I would start bleeding, and, like, I never consented to that.” Eirinn experienced an unwanted sexual act that resulted in bodily harm. Physical violence in our participants’ talk related to actions by ex-partners that could cause the participant some form of bodily harm. *Hitting*, *pushing against the wall*, and *scratching* were the most mentioned forms by our participants. Sexual violence was described in different ways, from *unwanted physically violent sex* to *transmitting sexual disease to a partner* or *having unprotected sex with others while hiding it from one's partner*. Although economic violence was construed as a form of abuse by several of our participants, they did not elaborate on this. Some related this to the fact that their partner “stole money” from them or that their partner would “deny them access to their own money” (Esin). Some illustrated economic violence in relation to a power imbalance between themselves and their abusive ex-partners.

Psychological violence, however, was the most predominant type of IPV that our participants defined. This finding resonates with previous research (Badenes-Ribera et al., 2015; Head & Milton, 2014). In addition to a higher prevalence rate, psychological abuse can lead to a higher rate of retrospective reporting because of the challenging nature of the symptoms, such as diminished self-esteem (Head & Milton, 2014). This may explain why participants predominantly define violence in terms of psychological abuse.

Our participants construed psychological violence in various ways, detailing how this kind of violence both reflects and feeds into the established power imbalance in the relationships, with the result that our participants’ well-being was severely damaged. One commonly mentioned form was *passive aggression*, understood by our participants as ignoring their partner's needs and emotions and pulling back. Passive-aggressive treatment could involve silent treatment, “using passive-aggressive eyerolling,” or “other ways of … making [the] other … feel bad without directly making them feel bad,” as Agnes puts it. *Manipulation*, *isolating*, and *mood swings* were other quite common themes in our participants’ accounts of psychological violence. *Gaslighting* as a form of severe psychological manipulation can be illustrated by the words of Eirinn, who describes how the abusive party “fucks with another party's perception of reality … [and] degrades or questions the other person's mental faculties.” Such manipulation may result in self-doubt and diminished belief in one's own judgment (Head & Milton, 2014). Gaslighting may indeed result in sustaining violence as the victim is constrained from coming to terms with the abuse they are exposed to, limiting their power to seek institutional protection (Sweet, 2019).

Several participants emphasized *control* as a sort of psychological violence. *Control* is construed as monitoring and limiting the activities of one's partner, especially social activities involving other people. For instance, Nadiya experienced this to the degree that their partner would be “dictating what [they] should wear, what should [they] do, who [they] socialize with [and] don’t socialize with.” Such control may lead to a form of alienation, stripping the person of individuality and agency (Dichter et al., 2018). Aru understands such control as “a violation of trust and privacy” and relates it to, for instance, jealousy by their partner: “They do it because they get really jealous… Like checking who you are with. And maybe there's an extra anxiety for lesbian relationships because women are naturally friends with other women, and so that complicates it.” Aru elaborates that jealousy can be particularly complicated in queer women's relationships, leading to a larger control issue and thus creating more anxiety for queer women.

Several of the participants emphasized *denigration* as a sort of psychological violence. Esin clearly states that “underestimating them, telling them ‘Oh, you became so fat’ is violence.” Descriptions such as *degrading*, *putting down*, and *making feel worthless* were used by our participants. Such attempts might be related to a dominance strategy by the abusive partners, who wish to gain more control over their partners. As Sinem illustrates, the abusive partner might, in their attempt at “making a person small,” come up with insults such as “you are not good enough, you are not smart enough … you cannot do that….”

### Making Sense of IPV

Our analysis produced five main themes that IPV experiences of our participants can be structured around: (a) power imbalance as a result of intersecting roles in immigration, racial/ethnic minority status, and heteronormativity; (b) difficulty acknowledging violence and feeling shame; (c) heterosexism and heteronormative discourse on IPV; (d) fighting back; and (e) childhood traumas, fear, and insecurity (Table 3).

**Table 3.** Making Sense of Experiencing Intimate Partner Violence.

Themes	Example quote
Power Imbalance: Intersecting Roles of Immigration, Racial/Ethnic Minority Status, and Heteronormativity	“If it comes to my options, the immigrant community is very heteronormative. And then, the others in the LGBTQ community are very white…. Well, the way I experienced it is that when you go into a group like that, it's very obvious that you are the different and more exotic person, with a different background and then it's kind of, like, sort of expected that you somehow explain it. I think it's what I, how I experience whiteness, it is just that it becomes apparent that you are different.” (Dilan, 27)
Difficulty Acknowledging Violence and Feeling Shame	“I feel like it's like kind of taboo to say that, or to admit kind of like accepting these things also, and especially to admit in a way like, this is hurting me and I am letting it happen, and then, like, people will hold you responsible, and, for your own thing or whatever.” (Eirinn, 26)
Heterosexism and Heteronormative Discourse on IPV	“In most queer relationships, the reasons to become violent are different. It's not about seeking dominance, and of making things how they should be according to society, but mostly because of growing up and seeing that you are something that is not to be accepted, something that is bad. You develop a lot of anger and trust issues that can come out in violent ways in a relationship.” (Elena, 25)
Fighting Back	“…I normally don’t shout, but if I feel like someone is being aggressive toward me then, then I get very aggressive.” (Aru, 29)
Childhood Traumas, Fear, and Insecurity	“You know, when you are in love, the problem is you don’t see it. You know that you are tired but you don’t see it, because you all the time think “I don’t want to be alone again.” (Yasemen, 37)

### Power Imbalance: Intersecting Roles of Immigration, Racial/Ethnic Minority Status, and Heteronormativity

As subjectivity—the way individuals think, feel, and act—is inevitably embedded in wider social and societal contexts, the IPV experiences of our participants are implicitly and explicitly influenced by those wider contexts. Indeed, all our participants refer to a power imbalance that “gives [their abusive partners] so much control over [their lives],” as Esin puts it, and they make the case that such imbalance may be related to, be strengthened and reproduced by socio-structural factors. More specifically, they grapple with the ways in which their immigrant status, given the immigration policies in Norway; their racial and ethnic background, given societal discourses regarding *race* and ethnicity; and their sexual orientation, given the heteronormativity of society, may feed into the overall experience of IPV. To begin with, we traced an explicit attempt by our participants at negotiating their immigrant background, illustrating how being a linguistic, cultural, or *racial* minority in a white and heteronormative society might be relevant to making sense of IPV *directly* and *indirectly*. Generally, representing a minority and thus being “different” from the norm in multiple ways is experienced as problematic:

If it comes to my options, the immigrant community is very heteronormative. And then, the others in the LGBTQ community are very white…. Well, the way I experienced it is that when you go into a group like that, it's very obvious that you are the different and more exotic person, with a different background and then it's kind of, like, sort of expected that you somehow explain it. I think it's what I, how I experience whiteness, it is just that it becomes apparent that you are different. (Dilan)

The intersectional perspective we use may help us illustrate the ways in which our participants experience problems. Dilan is forced into either a sexual minority position in an immigrant community that is predominantly heteronormative or a cultural/racial minority position in an LGBTQ community that is predominantly “white.” The intersectional problem lies in the fact that it is complicated to be gay in a heteronormative group, as it means risking prejudice, homophobia, and “being kicked out” by family and significant others (Agnes), and having to explain who you are as the “exotic other” (Dilan) adds to the strain. As Frankenberg (1993) argues, being white in predominantly white cultures is taken for granted to the extent that it becomes invisible, while nonwhites become visible minorities that need to be accounted for, explained, and problematized.

Merely being a visible minority may thus create a situation in which our participants cannot escape but face an implicit form of derogation. They may have to deal with feelings of inferiority and be exposed to racist discourse. According to Nadiya, “ethnic background makes everything more difficult.” While Eirinn, who had been exposed to various forms of physical, sexual, and psychological violence, had to also listen to “racist shit [their ex] had said and done” and “endure [their] racial abuse,” Sinem stated that their partner used their racial and immigrant background to degrade and humiliate them:

They were always telling me, “You only got this position because you’re brown.” Like, I was asked to talk about something in a panel, and they were like, “Cause you're brown.” Not because I’m this intellectual being … I’m never, like, an intellectual being ever in people's eyes … like I’m someone's brown experience. And yeah, it really fucked me up. (Sinem)

Such a racist discourse, where Sinem's subjectivity is reduced to merely a representative of a category, may not only strip her of all perceived intellectual abilities but also create psychological problems for her. There is indeed vast literature on how racism negatively influences psychological well-being (Harrell, 2000). For instance, Elgvin et al. (2013) documented how some LGBTQ people from ethnic minority backgrounds in Norway felt rejected by the mainstream gay community. Given the intersectionality regarding immigrant background, gender, and sexual orientation, some LGBTQ people with an immigrant background in Norway seem to have a higher risk of being marginalized (Bufdir, 2021), which might lead to a power imbalance among queer couples.

Esin, who came to Norway through marriage migration, narrated her IPV experiences as:

She gave me the feeling of that I was never [good] enough… Like how I can do this, and I did grow up in a village, and my race also, you know … she said, “You’re nothing to the Norwegian government and Norwegian police because, in the end, you are a black-headed Turkish woman. Who cares about you here if you get divorced from me? They will kick you out of the country.” That was kind of also the threats, like “I will call the police; they will come and take you.” (Esin)

Esin reported having been exposed to various forms of physical, economic, and psychological violence, involving instances of being pushed and hit, receiving silent treatment, body shaming, and humiliation, as well as different types of threats related to her immigration status and ethnic background. Her account illustrates well the intersectional experience, detailing the interrelatedness of different axes or forms of oppression and how they are mutually constitutive in creating a vulnerable subjectivity. Esin's precarious immigration status in Norway arguably became a constitutive element of her IPV experience. Talking about her migration status, she said:

I believe [it] effects the violence dynamics between two women also—like, power as economic right. One is owning the country, and one is not… It gives them so much control over your life, how you behave … it's about the power… (Esin)

Esin relates to and reflects on how her status as a migrant spouse creates a dependency on her Norwegian wife and thus a power imbalance in the relationship. Hence, structural, cultural, and economic elements of society may function in a way that places Esin in an underprivileged position while providing the abusive partner with a great deal of control over her life. This intersectional experience can be further illustrated by taking into account what Esin had experienced when she, as the foreign spouse, had sought help by calling the police to report severe physical violence:

I called the police to report domestic violence, and I asked them to come and get me and take me somewhere else because I was literally frozen, and I didn’t know what to do…. And I called them next to my ex-wife, I spoke with a police officer in English, and suddenly this police officer said, “Can I talk to your partner?” And I gave phone to my partner…. Then the police officer talked to my ex-wife, and they started to laugh, or she started to laugh, at least. And then she hung up the phone, and she said, “They don’t take you seriously because you can’t speak Norwegian.” (Esin)

Esin's account exemplifies the similar experiences among our participants. The fact that Esin came to Norway through marriage migration seems to establish a power imbalance between her and her Norwegian wife, given that her residential status in Norway had been dependent on her marriage. The socio-structural elements of Norwegian society, more specifically the bureaucratic rules around marriage migration with regulations to prevent pro-forma marriage, not only define a normative understanding of what a so-called *real* intimate relationship should look like but also generate a residential and economic dependence of the foreign spouse on the partner who already resides in Norway (Eggebø, 2010, 2013). We suggest that such dependency, that being the cultural and linguistic other, further disempowered our participant and became a constitutive part of her violent experience.

The ethnic minority background of our participants seemed to create certain vulnerabilities for them, as it could be used against them as a means of control by their abusive partners. It may be used against them as a threat, as Nadiya's case exemplifies: “I got threats that [I am] gonna be reported to immigration authority, if I do that and that.” Beyond serving as a direct means of control, being the migrant spouse in the relationship, as some of our participants reported, made them dependent on their partners psychologically as well as economically. Their dependency would vary according to “how long they had lived in Norway” and whether they had “personal income,” a “Norwegian support system” or network of friends they could rely on if needed, their “immigration statuses,” and a “permanent residence permit.” Previous research has emphasized that IPV, specifically victimization, affects queer people from racial/ethnic minority backgrounds disproportionately (Reuter et al., 2017; Whitton et al., 2019).

In addition to difficulties caused by ethnic minority status, heteronormativity as an overarching ideology of the society or an axis of sexual oppression may create further vulnerabilities for queer people, feeding into their experiences of IPV. Not being able to leave a violent relationship due to insecurities about being queer suggests that sexual minority status in a predominantly heteronormative society can structurally contribute to a higher degree of dependency in queer relationships.

I think also because there's a fear, maybe particularly in queer communities, and maybe queer with ethnic minority communities, that you cannot find someone to date, you know. There's a lot of fear that you’ll be alone, that you won’t have a family. It's hard to date, it's hard to find a companion. So, once you are in a relationship, you have a tendency to really want to hold on to that person, and that makes it even more vulnerable. Because it's harder for you to let go of a relationship once you’ve found it… (Aru)

### Difficulty Acknowledging Violence and Feeling Shame

Several participants stated that it takes time for them to recognize their experiences as violence. Agnes explains:

Things get like, really, really crazy. And you’re not able to realize it, what's going on… Even though you know something is not right, but still try to convince yourself and justify things… (Agnes)

Agnes’ process of realization, grappling with the abuse by searching for justification, may illustrate how difficult it might be to acknowledge and accept victimhood. A lack of a frame of reference related to IPV among sexual minorities may lead to difficulty acknowledging violence (Head & Milton, 2014). This process is perhaps complicated by the feeling of shame, as several of our participants state how difficult it is for them to be open about it and talk to others because it is experienced as “uncomfortable” and as “an embarrassment.” For instance,

I feel like it's like kind of taboo to say that, or to admit kind of like accepting these things also, and especially to admit in a way like, this is hurting me and I am letting it happen, and then, like, people will hold you responsible, and, for your own thing or whatever. (Eirinn)

Shame in same-sex relationships is frequently discussed in the context of IPV (Alsaker et al., 2016; Townsend & Bailey, 2021). Similar to the sense of shame related to the IPV in heterosexual relationships (Alsaker et al., 2016), queer women in our study also linked the feeling of shame to the fact that they were not able to leave their partners.

[I]t is quite embarrassing to admit something like that. To say I am in an abusive relationship and not leave. And it takes some time to understand that you are in abusive relationship… (Nadiya)

The feeling of shame related to IPV might lead the victims to social isolation and helplessness (Townsend & Bailey, 2021) and make queer women of ethnic minorities more vulnerable in the context of seeking help.

#### Heterosexism and Heteronormative Discourse on IPV

Queer women in our study mostly contend that the heavily predominant heteronormative thinking regarding violence in intimate relationships contributes to concealing queer experiences of violence. Sinem for instance makes the claim that from a heteronormative perspective:

When it comes to #metoo movements, there have been some, like, sexual violence or so much more, that is like, I have to prove my sexuality, because my sexuality doesn’t exist, and therefore, I can never be like the victim of sexual abuse, in a way. And so, then narratives are really, really bad, psychological violence is real, manipulation is real, but, and mainstream feminist will see all of these nuances when it comes to a man who's doing it to a woman, and we, we tried to create a hashtag, like, queer too, me too, you know, try to, but it didn’t catch on. (Sinem)

The quotation above illustrates how pervasive heteronormativity as an ideology or discourse might function as an alienation mechanism that invalidates, overlooks, and conceals various problems of queer existence. In a heteronormative society where the power imbalance is only normalized between men and women, the suffering of queer women who experience violence and forms of harassment perpetuated by other women is rendered invisible. Sinem argues that her experiences of violence in queer relationships are not recognized in a heteronormative society. She further raises a critique of the mainstream feminist movement that is not inclusive of issues regarding the IPV experienced in queer relationships. Reflecting on similar experiences, Aru stated that there is a mainstream belief that “women can’t be oppressors” and that they cannot be violent.

The argument that dominant heteronormative discourse might make violence experienced by queer women invisible is in line with established research (Irwin, 2008). Coupled with the stereotypical understanding of same-sex relationships as more gender-role free or more egalitarian (e.g., Hassouneh & Glass, 2008), heteronormativity, to the degree that it is appropriated/internalized, may lead queer women to understand violence as primarily a heterosexual issue and thus make it more difficult to acknowledge what they experience as violence and consequently stay silent about it (Irwin, 2008). Regarding this issue, Aru states:

I dated men before as well, and because they were men, I was even more aware, and if I saw any signs of a violent behavior then I would … like, leave immediately. But with women, it was more difficult for me to see because my view of women was much more positive and reassuring, and I felt safe … so it was harder to see those signs … I think we have this misconception, and I have this as well, where I always see myself as extremely considerate, but when I’m fighting with my partner, I see a different side of myself that I have. It took me a long time to accept that I have this side, that I can be the aggressor, and I can be manipulative, I can be all these things … (Aru)

Aru's reflection above relates to how internalization of heteronormative discourse on IPV by queer people might pose a huge problem, as it may contribute to concealing suffering by queer women (Hesselberg, 2011; Ovesen, 2020). Equally important, however, is the argument by some of our participants that IPV in queer relations should be contextualized and understood in terms of growing up in a heterosexist culture. Esin, for instance, contemplates:

I think it is so naive to say “Why a woman should hurt another woman? We are all woman. We should understand each other” … [b]ut we all grew up with heterosexual parents and mostly men power, then women can be like that, when they start to, when they are obliged to think like a man. I don’t mean like a man in a biological term…. Women can be so easily violent to each other, also… (Esin)

Esin elaborates on a widespread misconception about queer partnerships as egalitarian relationships. Referring to being brought up by heterosexual parents and exemplifying how women might be “obliged to think like a man,” she argues that a power imbalance inevitably generates a relationship dynamic which is heteronormative. However, how our participants relate IPV to heterosexism, which is conceptualized as societal devaluation of LGBTQ identities (Meyer, 2003), varies. Elena proposes another point of departure:

In most queer relationships, the reasons to become violent are different. It's not about seeking dominance, and of making things how they should be according to society, but mostly because of growing up and seeing that you are something that is not to be accepted, something that is bad. You develop a lot of anger and trust issues that can come out in violent ways in a relationship. (Elena)

Here, Elena construes violent behavior as a reflection or outcome of the continuous *othering* that queer people experience. Lack of acceptance of who they are and/or being valued negatively by the majority might underpin the IPV experience. Heterosexism arguably creates various distal (i.e., experiences of discrimination) and proximal (i.e., internalized heterosexism) minority stress processes for sexual minorities (Meyer, 2003). Indeed, there is support in the research literature, showing the existence of a relationship between internalized heterosexism and a higher risk of IPV (Balsam & Szymanski, 2005; Ummak et al., 2021). Consequently, we argue that our participants might be at higher risk of experiencing IPV given the exposure to such stressors and the degree to which they internalize them. Aru narrated:

My partner can’t meet my family, still hasn’t met my family, and it's like a part of you be[ing] excluded from the relationship. And that is a stressor, that's something that affects relationship dynamic … I think it brings out a lot of insecurities because you’re constantly on guard, and you’re thinking about what other people think about you. I mean, in general as well, just being, just being afraid in the streets, feeling unsafe as a woman but also as a lesbian woman. All these things, all these insecurities, it affects you in your day-to-day… it makes you more impatient, it makes you angrier, and it makes you sad and, you know, it definitely has an effect. If you happen to get in a conflict with your partner, then all of those factors contribute, even though you’re fighting about, you know, the bread, you’re actually, you’re other bringing into it a lot of other stressors. (Aru)

Aru's reflection above relates to the “personal is political” mantra widespread within critical segments of the social sciences, illustrating here that the dynamics of intimate relations are inevitably embedded within and shaped by how society functions. Thus, heterosexism may create LGBTQ-specific factors (connection to the LGBTQ community, lack of supportive networks, etc.) contributing to overall IPV experience for LGBTQ groups (see, for instance, Mason et al., 2014).

### Fighting Back

Although every participant in our study clearly identifies their ex-partner as the perpetrator and thus holds them responsible for the IPV they have experienced, some make the case that they themselves might have acted in ways that may be considered violent. For instance, Nadiya negotiates their role in IPV, acknowledging that they themselves were violent sometimes. This would involve manipulation or passive-aggressive behavior. However, they would not refer to themselves as perpetrators but rather frame their acts as *fighting back*. In this regard, the following account is important:

I would say that the character of that person was definitely waking the worse in me … If there was not so much provocation or manipulation on her side, then I wouldn’t have to, like, manipulate from my own side. But I’m definitely not a person that would do something violent … [I]n relationship with a person like that, I am not sure if it is just so easy to stay as an innocent observer or you just automatically become participant. You automatically kind of start to participate in this…. So, I wouldn’t say I was a perpetrator, but you’re creating some kind of toolbox for how to get out of this dramatic episode with the people like that. (Nadiya)

Similarly, Aru reflected on their own aggressivity as a reaction:

She grew up that way, and she had a lot of people that shouted at her, basically, and she's kind of adopted that behavior a bit, and I normally don’t shout, but if I feel like someone is being aggressive toward me then, then I get very aggressive. (Aru)

In the accounts above, our participants narrated their acts of violence as self-defense or a reaction to being at the receiving end of violence. On the one hand, it could be argued that they try to justify their own acts of violence as merely self-defense. On the other hand, it should be noted that developing a certain way of reacting—“a toolbox,” as Nadiya puts it—might be exactly what is needed to survive in a violent relationship.

#### Childhood Traumas, Fear, and Insecurity

In attempting to make sense of why IPV occurs in their relationships and why it may be difficult to escape it, such as by ending the relationship, our participants refer to childhood traumas, fear, and insecurities of both themselves and their abusive partners. Either party in the relationship having experienced childhood trauma, physical, or sexual abuse is construed by some participants to have a bearing on their adult relationships. What the person, either the abuser or the victim, brings into the relationship in terms of negative life experiences, such as childhood victimization, thus seems to present a greater risk for IPV. For instance, Aru tries to make sense of the violence they experienced by referring to the traumatic past and painful experiences she and her partner had:

She's also experienced a lot of violence in her upbringing. And we both have very different triggers, and we’ve come out of our situations in a very different way… And her past trauma and my past trauma is really affecting us, and we both of us have not gone consistently to therapist previously… (Aru)

In Aru's account, we witness a bridging between past traumas and IPV. Interestingly, she refers to a process of socialization in which her partner may have learned how to be an abuser as she herself was abused. Indeed, Lockhart et al. (1994) found that queer women who have experienced childhood victimization (i.e., emotionally abused) report more vulnerability to verbal and physical abuse in their relationships. In addition, some research indicates that abusers are more likely to have been victimized as children (Peterman & Dixon, 2003).

Several of our participants narrated that fear of losing their partner and hence being left alone may function to keep them in a violent relationship, especially for queer people, as also mentioned earlier. Fear of losing the partner might be so strong that one avoids bringing up problems or refuses to see them:

You know, when you are in love, the problem is you don’t see it. You know that you are tired but you don’t see it, because you all the time think “I don’t want to be alone again…” (Yasemen)

## Discussion

The aim of this study was to increase our understanding of how queer women from ethnic minority backgrounds perceive and experience IPV at the intersection of immigrant status, race/ethnicity, sexual identity, and sexual orientation.

As our analysis shows, our participants have a broad understanding of what IPV is, and they all have experienced certain types of abuse related to IPV—namely, physical, sexual, economic, and psychological. Overall, power imbalance in the relationship seems to be a crucial factor shaping the ways in which our participants have experienced IPV. An intersectional approach may fruitfully be utilized to discuss how this power imbalance is constituted and contextualized for queer women from ethnic minority backgrounds in Norway.

The findings together imply that intersecting identities relating to immigration, racial/ethnic minority status, and sexual/gender minority status create vulnerabilities and particular experiences of IPV. Understanding how our participants and others with similar backgrounds experience IPV demands addressing those intersecting identities in combination (see Cole, 2009). Simultaneously being excluded or discriminated against by the cultural ingroup on the basis of sexual orientation, and being excluded or discriminated against by the majority group on the basis of ethnicity, race, and/or sexual orientation, is the case for most of our participants. When the perpetuator of IPV is a majority member, endowed with and reproducing majority culture's prejudices, and the victim is a minority member who is excluded from a potentially protective environment, their cultural ingroup, then the experience of IPV is exacerbated as our analysis reveals. Hence, racism, heteronormative cultural discourses, and immigration status not only render IPV in queer relationships invisible but also affect the internal dynamics and power relations in queer relationships that can further increase the risk of IPV. From a minority stress perspective, we also know that paying attention to the added stress of being a member of more than one minority group or power hierarchy within a minority group (e.g., being a queer woman from an ethnic minority background) is very crucial to obtain a nuanced understanding of the IPV experiences among LGBTQ individuals (Finneran & Stephenson, 2014).

From an intersectional perspective, there is a growing literature that has shown that queer people with minority backgrounds experience racism within the mainstream white queer environment both in Norway (Eggebø et al., 2018) and other countries in Europe (El-Tayeb, 2012). They also encounter various instances of heterosexism and heteronormativity in their ethnic communities, which force them to hide their sexual identity. Even though Norway is a country known to be tolerant to sexual diversity where the rights of sexual minorities are legally recognized and protected, it is documented that heteronormativity still shapes the everyday experiences of, for instance, queer youth (Svendsen et al., 2018). Furthermore, heteronormativity is reproduced and exalted through a culture of homotolerance (Røthing & Svendsen, 2010). We suggest that this cultural context should be taken into consideration in making sense of the IPV experiences of our participants.

Our analysis illustrates how being a sexual as well as an ethnic minority may generate a significant power imbalance for queer women who have a relationship with an ethnic Norwegian (i.e., majority member of society). Some of our participants have experienced abuse connected to their precarious visa situation in Norway. They were, for instance, threatened by their partner, who told them that they would be deported from Norway if they ended the relationship. Thus, dependency created by fear of deportation informs their “choice” to remain in an abusive relationship. Although it has been suggested that conflicts related to dependency and autonomy in same-sex relationships may be exacerbated by power imbalances (e.g., West, 2002), studies on IPV among LGBTQ individuals racial and ethnic minorities are very scarce. Arguably, then, keeping the intersectionality of ethnicity, race, and immigration in mind, our analysis provides some much-needed insight into understanding how queer women with minority backgrounds may experience IPV due to power imbalance in their romantic relationships.

Our analysis also shows that there is a certain degree of difficulty in acknowledging the IPV experiences among our participants and that feelings of shame are often attached to IPV. We suggest that the strong assumption of equality in partners’ same-sex relationships (e.g., Hassouneh & Glass, 2008) generates the idea that power imbalance cannot exist between, for instance, two women. Yet, the intersectional experiences of our participants have illustrated what role power imbalance may play, as our participants accommodate various ethnic, migratory, economic, and family backgrounds, all of which might put them in a minority position. We also argue that the IPV experiences of queer women remain often invisible in a heteronormative society, which further makes it harder for queer women to recognize and name the abuse they encounter in their relationship.

Although some studies indicate that sexual minorities might have both received and perpetrated some type of IPV in their lifetime (Lewis et al., 2015; Messinger, 2017), research on IPV in LGBTQ individuals is scarce, and the idea of “mutual battering” in queer relationships is controversial (Peterman & Dixon, 2003). Without making any truth claims here, the *defense* rhetoric among our participants that their own violent behavior should be seen as a strategy to preserve their well-being when exposed to violence from their partners makes sense given an intersectional approach. The concept of mutual battering might indeed end up invalidating their victimhood, as it presupposes equal power relations between the partners (see also West, 2002). However, intersectionality implies that precisely how IPV is experienced by our participants should be considered, at least in part, through their ethnic, economic, and sexual minority positions. We hope that our findings will nuance the mainstream “mutual battering” argument in queer relationships.

Themes such as childhood traumas, fear, and insecurities are frequently discussed in the literature on IPV among heterosexuals (Grych & Kinsfogel, 2010). Our analysis, regarding the queer population, provides novel findings, revealing similarities in experiencing IPV in both queer and heterosexual relationships.

## Limitations

Several limitations should be mentioned. First, all interviews were done in English, which means that the recruitment process targeted queer women who can speak English. At the same time, the fact that the interviews were not done in the native language of the informants might have prevented them from expressing themselves adequately. Second, the sample size might be considered small. Reaching a specific minority section (identifying as cis-women and having a sexual orientation of lesbian, bisexual, pansexual, demisexual, etc.) within a minority group, LGBTQ populations, and with an immigrant background is indeed a difficult task. Thus, a caution is warranted regarding transferability of the analysis presented in this paper. However, leaning on, among others, Kvale and Brinkman (2009) and Trochim (2005), an argument for analytical generalization can be made that the analysis of the current study could be transferable, depending on similarities between time, place, people, and social contexts. For instance, as discussed above, some of our findings resonate with Hesselberg (2011) who also reported on IPV in lesbian relationships, pointing out that women can indeed be oppressors, and with Stubberud and Eggebø (2020) who reported about intersectional experiences of racism and heterosexism among queer immigrants in Norway. Third, our participants have predominantly identified their gender identity as cis women. Thus, the IPV experiences and their implications might be different for nonbinary and trans individuals. Lastly, we recruited participants with the help of announcements from LGBTQ organizations on their social media accounts, which means that the recruitment process targeted queer women who were more “out” and active in the community. Therefore, experiences and views of our participants may not reflect those of queer women who are less out or not associated with LGBTQ organizations and communities.

## Implications for Research and Practice

Our findings demonstrate that intersecting identities relating to immigration, racial/ethnic minority status, and sexual/gender minority status may create particular vulnerabilities related to IPV. Research is warranted with broader samples of queer individuals from more diverse backgrounds (e.g., varying gender identities, sexualities, age etc.), to expand on the findings of the current study. Given our findings, we further suggest that scales used to measure IPV are reviewed to ensure that they capture the specific types of violence experienced by LGBTQ populations with several intersecting minority identities.

Mental health professionals working with LGBTQ individuals will surely benefit from understanding how intersecting identities create the unique vulnerabilities of IPV. Such intersectional understanding may help them more readily identify the multi-axiomatic contextual factors underlying IPV and their role in maintaining mental health challenges among LGBTQ clients, thus possibly providing them with ways to address the adverse consequences of IPV. Mental health professionals should also advocate for social justice, by demanding improved policies and services related to LGBTQ-IPV, which might reduce the health disparities across all gender and sexual identities. Social justice-based therapies, which locate the problem in the societal context and not the individual, may be particularly helpful for this group (Myers et al., 2002).

## Conclusion

Though equality is a widely assumed characteristic of Norwegian society, government reports and our findings alike suggest that heteronormativity and racism do function as enhancive stressors among queer women of ethnic minorities within the context of IPV in Norway. We suggest, therefore, that not only should more work be done to make the problem of IPV among this group visible, but also psycho-social interventions should be tailored to address the specific needs of this group.
